# Kupffer Cells in Non-alcoholic Fatty Liver Disease: Friend or Foe?

**DOI:** 10.7150/ijbs.47143

**Published:** 2020-06-23

**Authors:** Jiajia Chen, Xiaoyi Deng, Yongjian Liu, Qiuhua Tan, Guidong Huang, Qishi Che, Jiao Guo, Zhengquan Su

**Affiliations:** 1 Guangdong Engineering Research Center of Natural Products and New Drugs, Guangdong Provincial University Engineering Technology Research Center of Natural Products and Drugs, Guangdong Pharmaceutical University, Guangzhou 510006, China.; 2 Guangdong Metabolic Diseases Research Centre of Integrated Chinese and Western Medicine, Guangdong TCM Key Laboratory for Metabolic Diseases, Key Laboratory of Modulating Liver to Treat Hyperlipemia SATCM, Level 3 Laboratory of Lipid Metabolism SATCM, Institute of Chinese Medicinal Sciences, Guangdong Pharmaceutical University, Guangzhou 510006, China.; 3 Department of Pharmacy, Affiliated Hospital of Guilin Medical University; 15# Lequn Road, Guilin, Guangxi Zhuang Autonomous Region 54101, China; 4 Guangzhou Rainhome Pharm & Tech CO., LTD 5F, No.10 Yongsheng Road, Yonghe Econoic region, Science City, Guangzhou 510663, China.

**Keywords:** non-alcoholic fatty liver, Kupffer cells, inflammation, lifestyle interventions

## Abstract

The prevalence of non-alcoholic fatty liver disease (NAFLD) is increasing all around the world and it may become the primary cause of terminal liver disease in adults and children in the next few decades. However, the pathogenesis of NAFLD is complex, and the Food and Drug Administration (FDA) has not approved any drugs for its treatment. Kupffer cells are the key cells regulating immunity in the liver, and the effect of their unique polarization on NAFLD has received increasing attention. Kupffer cells mainly reside in the lumen of hepatic sinusoids and account for 80% to 90% of colonized macrophages in the human body. They are phagocytic cells with the capacity for self-renewal that rarely migrate from their niche in the liver, and play a crucial role in regulating and maintaining homeostasis. Upon liver damage, Kupffer cells will be activated, releasing a good deal of inflammatory cytokines and chemokines. This review summarizes the multiple roles of Kupffer cells in the pathogenesis of NAFLD, the role of infiltrating macrophages in the pathogenesis of NAFLD is also briefly discussed, and aims to provide a theoretical basis for designing an NAFLD treatment strategy with Kupffer cells as the therapeutic target.

## Introduction

Non-alcoholic fatty liver disease (NAFLD) is a public health problem worldwide, and 25% to 30% of the global population suffers from different degrees of it 1. Due to the increasing prevalence of NAFLD, the clinical and economic burden of this disease is also increasing 2. Furthermore, a growing number of patients with cirrhosis and end-stage liver disease require liver transplantation, and the incidence of hepatocellular carcinoma (HCC) is expected to increase dramatically 3, 4. By 2030, the burden of late-stage liver disease in both Western countries and several Asian countries is estimated that increase 2-3-fold 5, 6.

**Figure 1:**
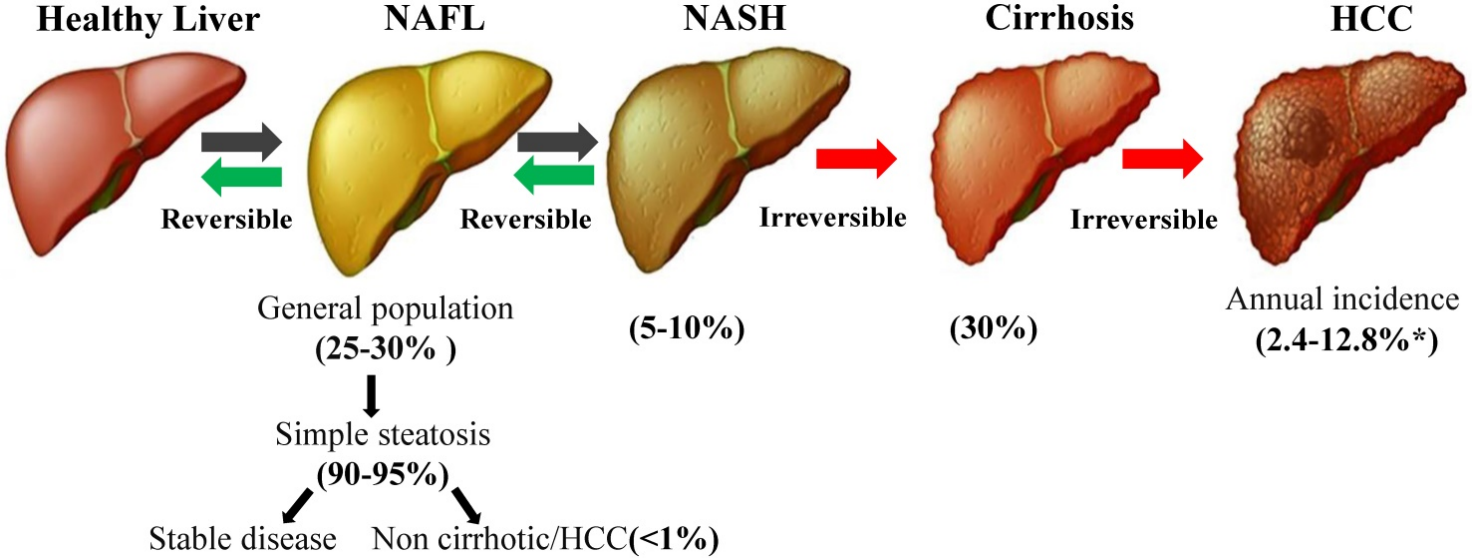
** The progression of NAFLD.** NAFLD covers a wide range of conditions, from simple fat accumulation (fatty liver or steatosis) to NASH, cirrhosis and HCC. Although the prevalence of NAFLD is very high in the general population, approximately 25-30%, the vast majority of patients present with simple steatosis, which not associated with survival or liver damage. Only 5-10% of patients with NAFLD develop NASH, and 30% of patients with NAFLD develop cirrhosis. *Depending on the disease state (with or without cirrhosis), the incidence of HCC may range from 2.4% to 12.8% 118. NAFLD: non-alcoholic fatty liver disease; NASH: non-alcoholic steatohepatitis; HCC: hepatocellular carcinoma.

As the name implies, NAFLD occurs without excessive alcohol consumption, which is defined using the widely used high alcohol intake thresholds of 20 g/day for women and 30 g/day for men 7. The hallmark pathological feature of NAFLD is the accumulation of triglycerides in the cytoplasm 8, and the gold standard for diagnosing the presence and severity of NAFLD is a liver biopsy 9. The spectrum of histological findings in the liver attributed to NAFLD includes simple steatosis without obvious signs of inflammation or fibrosis [non-alcoholic fatty liver (NAFL)], hepatic triglyceride accumulation with inflammation and hepatocyte damage (ballooning) [non-alcoholic steatohepatitis (NASH)], liver fibrosis and cirrhosis and/or HCC 10-12 **(Figure 1)**. Moreover, NAFLD is also a systemic disease related to obesity, type 2 diabetes mellitus (T2DM), insulin resistance (IR), cardiovascular disease (CVD), and metabolic syndrome (MetS) 13, 14.The pathogenesis of NAFLD is a multistep process that begins with triglyceride accumulation and leads to hepatic steatosis, which was originally proposed in the “two-hit hypothesis” 15. According to this hypothesis, the second hit is the induction of oxidative stress and inflammation, which promote pathological progression 16. In fact, the pathogenesis of NAFLD, particularly in the smaller subgroup of patients with hepatocyte injury, inflammation and/or fibrosis, is not completely understood 17.

In the past 20 years, numerous studies of basic pathology and clinical trials have been conducted worldwide to better understand and prevent the development of NAFLD, but the Food and Drug Administration (FDA) has been approved no drugs. Currently, the general therapeutic strategies for NAFLD are as follows: (1) implementation of lifestyle interventions, including exercise and a proper diet; (2) active treatment of MetS to improve IR, weight loss, blood pressure and dyslipidemia; (3) treatment of liver injury to inhibit oxidative stress and improve liver fibrosis; and (4) perform liver transplantation in patients with advanced end-stage liver disease. Considering the increasing clinical burden and poor quality of life of patients with NAFLD, studies aiming to identify effective strategies that slow the increase in the number of NAFLD cases and improve the therapeutic options are urgently needed.

## Origin and classification of hepatic macrophages

Hepatic macrophages mainly consist of two types: Kupffer cells, which originate from yolk sac-derived red bone marrow progenitor cells and reside in the liver, and monocyte-derived macrophages, which are derived from bone marrow hematopoietic stem cells and transplanted through the blood circulation to the liver 18. Essentially, monocyte-derived macrophages are immunogenic macrophages that can differentiate under the influence of microenvironment; Kupffer cells are a self-sustaining, locally proliferating, and usually susceptible population of phagocytic cells 19. More importantly, a recent study reported differences in the transcriptome between infiltrating macrophages and Kupffer cells in NASH mouse models, and the expression of certain genes was significantly different; for example, 1500 genes were enriched in infiltrating macrophages, and 1690 genes were enriched in Kupffer cells 20. In general, Kupffer cells are able to be distinguished from monocyte-derived macrophages. CD68, a scavenger receptor (SR) for lipoproteins on macrophages is used to indicate the presence of macrophages in the liver 21. Moreover, Kim et al. performed dual staining of mouse liver section using ionized calcium binding adapter molecule 1 (IBA-1) and C‐type lectin domain family 4 member F (CLEC‐4F), which adequately differentiated between infiltrating macrophages and Kupffer cells 22.

In addition to the above-mentioned hepatic macrophages, other macrophages also play more and more roles in the pathogenesis of NAFLD. For example, adipose tissue macrophages (ATM) promote the recruitment of blood mononuclear macrophages to the liver during the occurrence of NAFLD, and the activation of adipocyte death-related macrophage may promote liver injury by stimulating neutrophil recruitment, thereby exacerbating lipolysis-mediated lipotoxicity and liver injury 22, 23. When Nod-like receptor protein 3 (NLRP3) inflammatory bodies are activated, they promote macrophage activation in subjects with NAFLD and accelerate liver inflammation 24. In high fat diet (HFD) fed mice, the neutrophil‐derived reactive oxygen species and activation of stress kinases lead to the hepatic overexpression of C-X-C motif chemokine ligand 1 (CXCL1) and drive the progression of steatosis-to-NASH 25. In summary, macrophages play an essential role in maintaining homeostasis and defense functions.

## Topology and polarization of Kupffer cells

Kupffer cells are the resident liver macrophages and account for 80-90% of all macrophages in the body. They are the largest component of the reticular endothelial system 26 and represent a resident self-renewing phagocytic population in the liver. Kupffer cells migrate between liver sinusoids and the space of Disse, directing and regulating interactions between various resident and recruited cells in the liver 27. It was found that Kupffer cells lost in the early stages of the mouse NASH model fed by a methionine-choline-deficient (MCD) diet, and then the Ly6C^+^ monocyte-derived macrophages replacing the resident Kupffer cells as the dominant macrophage population 28. Kupffer cells express Toll-like receptor (TLR) 4 and bind to lipopolysaccharide (LPS), resulting in the activation of nuclear factor-kappa B (NF-κB), mitogen-activated protein kinase (MAPK), extracellular signal-regulated kinase 1 (ERK1), p38, Jun N-terminal kinase (JNK) and interferon regulatory factor 3 (IRF3); subsequently, the production of large amounts of pro-inflammatory cytokines leads to liver damage, leukocyte infiltration and the activation of hepatic stellate cells 29.

Historically, macrophages have been considered to undergo phenotypic differentiation in response to different microenvironmental signals, and the phenotypes have been traditionally oversimplified as classically activated macrophages (M1) and alternatively activated macrophages (M2) 30, 31. M1 macrophages are induced by pro-inflammatory mediators, have a high antigen presentation capability, and induce the release of large amounts of cytokines, such as tumor necrosis factor (TNF), inducible nitric oxide synthase (iNOS), interleukin (IL)-1β, and reactive oxygen species (ROS) while simultaneously activating the Th1 response. In addition, M1 macrophages exert anti-proliferative and cytotoxic effects, and promote macrophage-mediated tissue damage. In contrast, M2 macrophages show anti-inflammatory and pro-degradation activity, such as arginase 1 expression, IL-4 and IL-10 secretion and high levels of phagocytosis 32, 33. M2 macrophages are further classified as M2a, M2b, and M2c and each subtype of cells secretes different cytokines and expresses different epitopes. M2 macrophages, which balance the activity of M1 macrophages, are involved in downregulating the inflammatory response and initiating tissue repair, and these cells synthesize important mediators that promote tissue remodeling and vasculogenesis to control the inflammatory response; these cells also promote the Th2 immune response and play a key role in regulating T cell function 34, 35.

**Figure 2:**
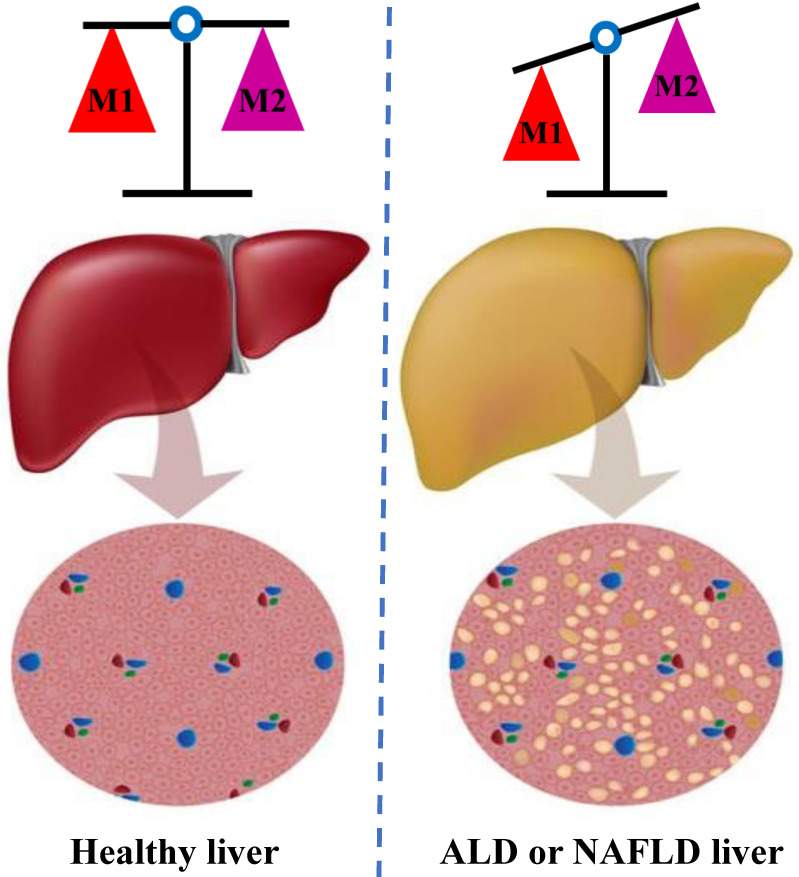
** The balance of M1 and M2 Kupffer cells.** Kupffer cells maintain a balance between the M1 and M2 phenotypes to control inflammation in individuals ALD and NAFLD. M1 Kupffer cells secrete a large amount of pro-inflammatory factors, such as IL-1β, TNF-α and IL-6; in contrast, the M2 Kupffer cells secrete a numerous anti-inflammatory factor, such as IL-4and IL-10. M2 Kupffer cells balance the activity of M1 Kupffer cells, initiate tissue repair, synthesize important mediators to promote tissue remodeling and angiogenesis, and control the inflammatory response. ALD: alcoholic liver disease; NAFLD: non-alcoholic fatty liver disease. IL: interleukin; TNF: tumor necrosis factor.

In individuals with alcoholic liver disease (ALD) and NAFLD, the balance between classic pro-inflammatory M1 Kupffer cells and selectively activated anti-inflammatory M2 Kupffer cells is crucial to strictly regulate the occurrence and development of liver injury 36 (**Figure 2**). Through *in vivo* and *in vitro* experiments, J. Wan and colleagues showed that the selective induction of M1 Kupffer cells apoptosis enabled M2-polarized Kupffer cells to protect against alcoholic liver injury by neutralizing the remaining M1 Kupffer cells; these researchers concluded that limiting the number of M1 Kupffer cells while promoting M2 Kupffer cells polarization may be a valuable therapeutic strategy for ALD 37. Moreover, strategies that promote the polarization of anti-inflammatory M2 Kupffer cells prevented alcohol-induced steatosis and hepatocyte apoptosis, and IL-6 is a mediator of hepatocyte senescence induced by M2 Kupffer cells 38. Interestingly, the occurrence of NAFLD/NASH is often accompanied by an increase in the proportion of M1/M2 macrophages in the liver tissue 39. Experiments conducted in a mouse model of NAFLD further showed that M2 macrophages induce the apoptosis of M1 macrophages by secreting arginase and the anti-inflammatory factor IL-10, which act in a paracrine manner; in addition, these M2 macrophages reduced the proinflammatory effects of M1 macrophages and then mitigated the inflammatory response and cell damage 40. In summary, Kupffer cells exert various regulatory effects on different stages of liver injury and repair. The existing evidence implies that therapeutic interventions targeting M2 Kupffer cells polarization may be an appealing strategy for limiting inflammation and hepatocyte injury in the initial stages of ALD and NAFLD.

## Roles of activated Kupffer cells in NAFLD

Kupffer cells, with their high heterogeneity and plasticity, are excellent at maintaining homeostasis and defense functions. In the body, factors such as lipids, lipids metabolites, and LPS, activate Kupffer cells. Once activated, Kupffer cells increase the expression of inflammatory cytokines, exacerbate the degree of necrotic inflammation among liver cells, and alter the expression of genes associated with fibrosis and oxidative damage, leading to the development of NAFLD. The role of Kupffer cells in NAFLD, as elucidated in recent years, is summarized in four pathological processes, including inflammation, steatosis, oxidative stress, and fibrosis, which are discussed to provide a theoretical basis for designing treatment strategies for NAFLD with Kupffer cells as the therapeutic target.

### Inflammation

#### Inflammation and the gut-liver axis

The intestinal barrier acts as the body's first line of defense to protect against damage caused by bacteria, food antigens, toxins and other factors in the intestinal cavity 41. The liver, as the body's second line of defense, further regulates the immune function of the intestinal barrier. Together, the liver and intestinal barrier forms the body's defense system as the gut-liver axis 42. The pathogenesis of NAFLD, a multisystem disease, is related to the permeability of the intestinal barrier. The intestinal flora and related metabolites directly penetrate the intestinal barrier, enter the blood circulation and reach the liver, where they elicit an inflammatory response, and promote and exacerbate the development of NAFLD. For example, intestinal endotoxins (such as LPS) stemming from flora disorders, bacterial transmigration or changes in the intestinal mucosal barrier play a large role in the pathogenesis of NAFLD 43, 44. LPS is one of the most potent activators of the mammalian immune system and is released from the surface of bacterial cells during proliferation, lysis and death 45.Obese individuals or patients with NAFLD present with intestinal bacteria overgrowth compared with normal-weight or unaffected people, which results in an increase in the number of bacteria producing LPS 46, 47. Upon accumulation, LPS binds to Kupffer cell surface receptor complexes, leading to the production of a good deal of factors and the induction of immune responses and inflammatory reactions, thereby facilitating liver inflammation and pathological damage. The detailed mechanism is described below.

LPS binds to CD14 on the surface of Kupffer cells through the TLR4 signaling pathway, which subsequently recruits T lymphocytes, B lymphocytes and other leukocytes, resulting in the release of inflammatory cytokines and chemokines, such as TNF-α, IL-1β, IL-6, IL-12 and interferon-γ (IFN-γ) 48, 49. Next, a large multiprotein complex containing myeloid differentiation factor 88 (MyD88), TNFR-associated factors (TRAFs), IL-1 receptor-associated kinases (IRAKs) and transforming growth factor (TGF)-beta-activated kinase 1 (TAK1) forms 50, 51. Activated TLR4 is also translocated to an endosomal compartment, which results in the recruitment of TIR domain-containing adaptor inducing IFN-β (TRIF), RNA modifying proteins containing the conserved TRAM domain, TNF receptor-associated factor 3 (TRAF3) and other proteins 52. These events induce the phosphorylation of IRF3 and the IFN response 53. Activated Kupffer cells produce TNF-α, IL-6 and other inflammatory factors, recruit infiltrating inflammatory cells, and play multiple roles in NAFLD 54.

#### Inflammation and pyroptosis

Based on accumulating evidence, the liver is exposed to various microbial molecules produced in the digestive tract through the local circulation and can be infected by various hepadnaviruses, leading to the activation of inflammatory bodies and pyroptosis, which mediates the inflammatory defense mechanism against pathogens. During the pathogenesis of NASH, the levels of NLRP3 inflammatory bodies in the intestine are reduced, which weakens the intestinal barrier function against bacterial translocation, allowing a large amount of LPS to enter the liver and exacerbating the development of NASH 55.

**Figure 3:**
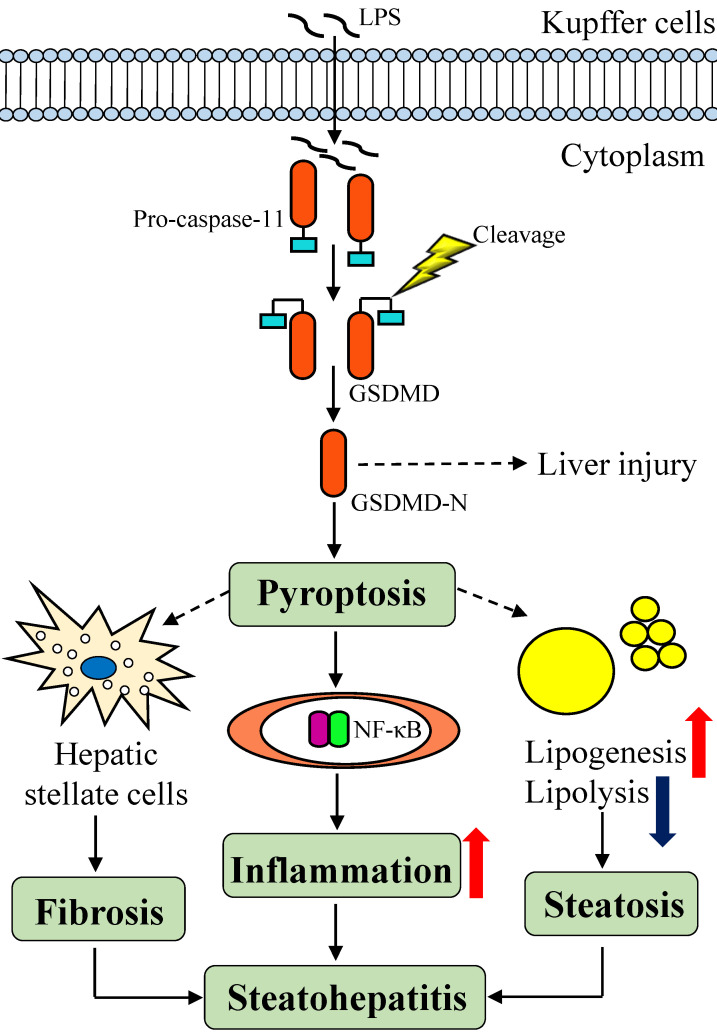
** The LPS-induced signaling pathways in Kupffer cells and mechanisms of GSDMD-induced pyroptosis.** LPS is sensed in a TLR4-independent manner in the cytoplasm through a directly interaction with caspase-11. Then, caspase-11 cleaves and activates GSDMD, which promotes pyroptosis. GSDMD acts as a direct executor of pyroptosis by causing increased production of in pro-inflammatory cytokines, and indirectly activates the NF-κB signaling pathway and subsequent macrophage recruitment. GSDMD also contributes to steatohepatitis by increasing lipogenesis and decreasing lipolysis. At the same time, pyroptosis activates hepatic stellate cells to induce liver fibrosis and even the death of hepatocytes in individuals with NASH. LPS: lipopolysaccharides; TLR4: toll-like receptor 4; GSDMD: gasdermin D.

One study revealed a new mechanism by which macrophages are activated by cytoplasmic LPS via a TLR4-independent pathway, thereby activating the inflammatory enzyme caspase-11 56 (**Figure 3**). As shown in the study by Shi and colleagues, caspase-11 exposes the N-terminal fragment of gasdermin D (GSDMD), which triggers an inflammatory cell death response called pyroptosis in response to cytoplasmic LPS that is essential for endotoxic shock in mice 57, 58. As the executor of pyroptosis, caspase-cleaved GSDMD directly increases the production of pro-inflammatory cytokines, indirectly activates the NF-κB signaling pathway and recruits macrophages 59. In this context, the discovery that GSDMD both indirectly and directly promotes lipid production through signaling pathways provides a new insight into the associated mechanism. Pyroptosis is correlated with NASH-associated hepatic fibrosis, which activates hepatic stellate cells to induce liver fibrosis and even hepatocyte death 60, 61. Bing Xu and colleagues analyzed human NAFLD/NASH liver tissues and showed that the levels of GSDMD and the pyrophosphorylation-induced fragment GSDMD-N were both increased, and more importantly, hepatic levels of the GSDMD-N protein were significantly increased patients with NASH and were related to NAFLD activity scores and fibrosis 62. Although the role of GSDMD in the pathogenesis of NAFLD and the potential underlying mechanisms remain unclear, GSDMD represents a potential therapeutic target or biomarker of disease progression for human NAFLD that deserves further attention in the future 63. The development of compounds or biopharmaceuticals that target cell death and inflammatory bodies represents an important approach for treating NAFLD /NASH.

### Steatosis

The liver has a high capacity for fatty acid uptake and plays a significant role in the metabolism of lipids and lipoproteins 64. The biological activity of Kupffer cells in individuals with NAFLD may be regulated by different mechanisms, such as the lipid abundance and composition and changes in the liver tissue 65. In a marginal-copper high-fructose diet (CuMF) rats, Kupffer cell ablation decreases hepatic triglyceride accumulation and plasma macrophage chemotactic protein 1 (MCP-1) level, suggesting that Kupffer cells protect liver cells lipid metabolism disorders and steatosis 66.

As an early stage of NAFLD, hepatic steatosis occurs when an imbalance exists between the inward and outward fluxes of free fatty acids (FFAs) in the liver and is defined as triglyceride accumulation in more than 5% of hepatocytes 67. Asanuma et al., performed superparamagnetic iron oxide (SPIO) magnetic resonance imaging and reported a significantly impairment in the phagocytosis activity of Kupffer cells in patients with NAFLD and NASH animal models without a reduction in cell number, and the degree of functional impairment was positively correlated with hepatic steatosis 68. Thus, the TLR-mediated recognition of fatty acid groups is a primary mechanism of lipid regulation in inflammation and innate immunity 69. In the Kupffer cell extracellular matrix, the binding of FFAs to TLRs activates the C-JNK and NF-κB pathways 70. Activated NF-κB upregulates the levels of adhesion molecules and MCP-1, thus recruiting CD11b^+^ macrophages and promoting lipid synthesis, which increases the transcription of activating protein 1 (AP-1) and pro-inflammatory cytokines 71. Excess FFAs that accumulate in Kupffer cells impair not only β-oxidation but also other mitochondrial functions 72.

Interestingly, increasing experimental and clinical evidence suggests an association between the pathogenesis of NASH and changes in intrahepatic cholesterol homeostasis and the accumulation of free cholesterol 73, 74. Rats fed choline-deficient (but methionine-sufficient) and high-cholesterol (2%) diets suffer from impaired mitochondrial function, which characterizes were the accumulation of free cholesterol and the depletion of glutathione, and increased susceptibility to TNF-α and fatty acid synthase (Fas)-mediated liver damage 75. Cholesterol synthesis pathways and hepatocyte uptake must be perfectly balanced with cholesterol clearance by hepatocytes to maintain liver cholesterol homeostasis 76. However, when Kupffer cells lack the cholesterol biosynthesis capacity, they must obtain cholesterol from circulating cholesterol-rich lipoproteins. The main cholesterol uptake pathway in Kupffer cells involves oxidized low-density lipoprotein (oxLDL) internalization by the SR clustering protein CD36 and SR-A. In patients with MetS or NASH, plasma oxLDL levels are significantly increased, particularly after a meal, and the SR-mediated uptake of these lipoproteins may be a major cause of Kupffer cells activation and inflammatory factor infiltration in the liver 77, 78. Consistent with these findings, strategies targeting defective oxLDL uptake secondary to the inactivation of Kupffer cells by reversing the genetic deletion of lipoprotein receptors or administering anti-oxLDL antibodies restore the resting Kupffer cells phenotype, reverse liver inflammation and reduce the incidence of NAFLD 79, 80.

### Oxidative stress

A common feature of chronic liver disease is oxidative stress, which affects the development of NASH 81. By observing changes in various oxidative stress markers in liver biopsies or blood samples, researchers observed elevated levels of oxidative stress in patients with NAFLD, and this level was related to the severity of NAFLD 82. Absent or dysfunctional FFA β-oxidation leads to the generation of ROS, which induce mitochondrial lipotoxicity and activate the NF-κB/JNK pathway, high mobility group box 1(HMGB-1)/TLRs, cytokines and chemokines 83, 84. In the liver, the main source of ROS is Kupffer cells, in which ROS are usually produced by nicotinamide adenine dinucleotide phosphate (NADPH) oxidase 85. Since the discovery of uncoupling protein 2(UCP2), this protein has been considered one component of the pathogenic mechanisms of NAFLD 86. According to previous reports, genetically obese (ob/ob) mice fed a high-fat diet show a significant enrichment of UCP2 in hepatocytes and a reduction in the numbers of Kupffer cells and other macrophages 87, 88. As described below, the overexpression of UCP2 inhibits ROS production and the activation of Kupffer cells and other macrophages; however, the inhibition or ablation of UCP2 leads to increased ROS levels and pro-inflammatory cytokine release and the continued activation of NF-κB 89, 90.

Oxidases in different cellular compartments, such as mitochondrial, NADPH oxidase and cytoplasmic xanthine oxidase, inhibit ROS production and damage the antigenicity of Kupffer cells to a similar extent 91. Moreover, because of the inability of UCP2-/- macrophages to control the mitochondrial ROS production, these cells show markedly increased antimicrobial and pro-inflammatory activities 92. Interestingly, any cell-derived ROS may affect the antioxidant defense and redox-sensitive signaling pathways in Kupffer cells. Fortunately, hepatocytes have a higher antioxidant capacity than Kupffer cells, and thus ROS-mediated regulatory mechanisms are thought to exert a greater effect 93. In addition, ROS and other oxidative stress mediators, such as the reactive aldehyde end products of lipid peroxidation, are postulated to maintain fibrosis progression in individuals with chronic liver diseases of different etiologies by inducing cirrhosis, which triggers cell damage and death, as well as fibrosis and inflammatory reactions 94. This approach may be a valuable avenue for future research on Kupffer cells.

### Fibrosis

During steatohepatitis, activated Kupffer cells secrete a large number of pro-inflammatory cytokines, such as TNF-α and IL-6, to recruit T lymphocytes, natural killer T (NKT) cells and other inflammatory cells, indirectly participating in inflammation and the immune response 95. NKT cells release perforin and granulase from fine particles that damage liver cells. The recruitment of NKT cells also promotes the activation of hepatic stellate cells and myofibroblasts, leading to the progression of NASH-associated fibrosis 95. In addition, lipid peroxides hydrolyze apolipoprotein B100, aggravate inflammatory necrosis, activate Kupffer cells, and induce the secretion of TGF-β1, an important anti-proliferative and profibrotic cytokine. Then, the TGF-β signal is transmitted through the TGF-β1 receptor (TβR) at the cell surface to promote the phosphorylation of the Smad protein in the nucleus, which leads to the transformation of hepatic stellate cells into muscle fiber cells and aggravates liver fibrosis 96, 97.

### Summary

The above description clearly highlights the multiple functions and various regulatory activities of Kupffer cells in the proregression of NAFLD and their important roles in pathological processes (**Table 1**). During the inflammatory response, LPS binds to CD14 on the membrane and signals through the TLR4 pathway to activate Kupffer cells and induce the production of various inflammatory factors; LPS also directly binds to Kupffer cells and activates the inflammatory enzyme caspase-11, causing pyroptosis and the release of inflammatory factors, highlighting the multiple roles of this molecule. During steatosis, FFAs mediate Kupffer cells activation and increased MCP-1 expression to promote lipid accumulation. During oxidative stress, Kupffer cell activation and ROS production form a positive feedback loop. In the process of fibrosis, Kupffer cells produce cytokines such as TGF-β1 and IL-6 to promote fibrosis.

## Intervention and treatment

### Lifestyle interventions

Currently, the FDA has been approved no drugs for the treatment of NAFLD/NASH. Lifestyle improvements, weight loss and increased exercise are the currently available and effective non-pharmacological treatments for NAFLD/NASH 98, 99. Comprehensive lifestyle changes, including a reduction in energy intake and an increase in physical labor, improve liver enzyme levels [alanine aminotransferase (AST), aspartate aminotransferase (ALT) and gamma-glutamyl transpeptidase (GGT)], metabolic parameters (fasting plasma glucose level and insulin sensitivity), and the triglyceride concentration in the liver [via proton nuclear magnetic resonance (NMR) spectroscopy] in 6 to 12 months 100, 101. These interventions have also been shown to improve steatosis and necrotic inflammation, including swelling and fibrosis, in paired liver biopsies 102, 103. Dietary interventions improve glucose and lipid metabolism in patients, reduce the body weight of obese patients, reduce IR, improve insulin sensitivity in peripheral tissues, and promote glucose uptake and utilization by muscle cells. Exercise increases energy consumption, reduces the conversion of energy into fat, reduces body mass, excite sympathetic nerves, increases insulin secretion, promotes fat decomposition, and reduces the occurrence of visceral obesity. Exercise also increases high-density lipoprotein levels and insulin sensitivity, thereby reducing the degree of fatty liver. Therefore, the formulation of scientific evidence-based exercise programs and the mastery of reasonable dietary principles are very important for reducing the morbidity of fatty liver disease, improving the reversal rate and quality of life of people with fatty liver disease, and reducing the burden of fatty liver disease on human health.

### Strategies targeting Kupffer cells

Kupffer cells are phagocytes with the capacity for self-renewal that rarely migrate from their niche and targeting Kupffer cells for the treatment of NAFLD has great significance. In an experimental NASH model established in mice, Rivera et al., found that disrupting Kupffer cells reduces histological manifestations in the liver, such as steatosis, inflammation, and necrosis 104. Similarly, Zeng et al., reduced the number of Kupffer cells with Gdcl3 in a high-fat diet mouse model and observed significant reductions in steatosis and IR 105. Therefore, strategies targeting Kupffer cells are an important aspect of NAFLD treatment (**Table 2**).

**Kupffer cells activation:** As mentioned above, activated Kupffer cells show increased expression of inflammatory cytokines and thereby exacerbate the degree of necrotic inflammation of liver cells, leading to the occurrence of NAFLD. The inhibition of Kupffer cell overactivation may reverse the course of this disease. Benzyl isothiocyanate (BITC) is an organic sulfur compound that is abundant in cruciferous vegetables. Recently, Chen et al. showed that BITC ameliorates the effects of a high-fat/high-cholesterol diet by inhibiting cholesterol crystal-activated NLRP3 inflammasomes in Kupffer cells, thereby preventing the development of diet-induced NASH 106. Alagebrium was shown to impair the progression of NAFLD in mice fed a diet containing advanced glycation end products (AGEs) by directly inhibiting Kupffer cells and indirectly inhibiting hepatic stellate cell activation; Alagebrium increases AGEs clearance and may inhibit the progression of NAFLD to liver fibrosis 107. In addition, the natural polyphenolic compound resveratrol (RSV) is considered to possess a wide range of bioactivities that are beneficial to human health. According to Takaomi et al., RSV improvs the inflammation and fibrosis in mice with HFD-induced NAFL by inhibiting LPS reactivity controlled by CD14 expression in Kupffer cells 108.

**M1 and M2 Kupffer cells polarization:** Kupffer cells are the key cells regulating hepatic immune responses, and their specific polarization has received increasing attention in research on NAFLD. In one experiment of mice fed the antioxidant β-cryptoxanthin, the total population of hepatic macrophages and T cells was reduced, but the population of M2 macrophages was increased. β-Cryptoxanthin may at least partially prevent and reverse IR and steatohepatitis by activating M2 macrophages/Kupffer cells 109. Similarly, Ni et al. proposed that dietary carotenoids, such as β-cryptoxanthin and astaxanthin, prevent or improve NAFLD by regulating macrophage/Kupffer cells polarization and hepatic homeostasis 110. As a key regulator, retinoic acid-associated orphan receptor α (RORα) can regulate M1/M2 polarization in Kupffer cells; RORα enhances the M2 polarization of Kupffer cells by inducing Kruppel-like factor 4 and may be a vulnerable therapeutic target in liver macrophages for the treatment of NASH 111. In recent years, the repurposing of old medicines has received considerable attention. For example, Li et al., found that liraglutide, the first-line drug for T2DM, regulates the M2 polarization of Kupffer cells through the cAMP-PKA-STAT3 signaling pathway, thereby reducing liver steatosis caused by a HFD 112. Analogously, Yang et al., found that Saxagliptin, a hypoglycemic drug, regulates the polarization of M1/M2 macrophages/Kupffer cells through the CaMKKβ/AMPK pathway, thereby reducing NAFLD 113.

## Conclusions and Prospects

In the past few years, our basic understanding of Kupffer cells has substantially improved Kupffer cells are a “double-edged sword”; they promote inflammation that leads to liver cell damage and activate hepatic stellate cells, causing fibrosis, angiogenesis and tumor development, but they also stimulate fibrosis regression and limit disease progression. Important issues to consider in future research include controlling the ratio of M1 to M2 Kupffer cells and therapeutically interfering with the M2 polarization of Kupffer cells. Although pyroptosis has been shown to be closely related to the progression of liver disease, many details remain unknown, and further research is required. For example, researchers have not clearly determined how pyroptosis causes liver damage and affects disease progression. Currently, more systematic research is needed to clarify the function of Kupffer cells in NAFLD and resolve the controversy. These studies will not only facilitate the further elaboration of NAFLD pathogenesis but also provide new research ideas and targets for NAFLD in the near future.

In recent years, research on the use of natural drugs in the treatment of NAFLD has expanded. Some herbs also have potential value as treatment for NAFLD. According to modern pharmacological studies, Salvia miltiorrhiza promotes blood circulation, improves the microcirculation, inhibits collagen fiber hyperplasia, and exerts anti-fibrotic, anti-free radical and anti-peroxidation activities 114, 115. The stilbene component of Polygonum multiflorum improves the function of damaged fatty livers in rats and reduces lipid peroxide contents in the liver. Moreover, P. multiflorum is rich in phospholipids that promote cholesterol and lipids metabolism in the liver, thereby reducing intrahepatic fat accumulation 116, 117. Perhaps further in-depth research will determine whether the aforementioned herbs slow the inflammatory response by regulating Kupffer cells and other macrophages to achieve anti-oxidant and anti-liver fibrosis effects.

In general, the therapeutic effects of certain drugs mentioned above on patients with NAFLD are controversial or unclear. The prevention and treatment of NAFLD, a metabolic disorder caused by an unhealthy lifestyle, must not rely solely on medication. A reasonable recommendation is to improve detrimental lifestyles and poor eating habits, increase aerobic exercise, and control body weight. The combination of lifestyle interventions and drug therapy is the basis for the treatment of NAFLD, and the efficacy of therapeutic drugs will depend on the success of lifestyle interventions.

## Figures and Tables

**Table 1:** The role of Kupffer cells in the various pathological processes of NAFLD

Pathological process	Activated pathway	Resulting effects
Inflammation	LPS binds CD14 via TLR4 pathway to activate IKK	Secretes inflammatory factors: TNF-α: involved in IR, promotes lipid synthesis, induces hepatocyte apoptosis IL-6: improves hepatic microcirculation and enhances immunity TL-1β: promotes lipid synthesis and accumulation IL-12: promotes the differentiation of Th1 cells and participates in NKT cell depletion
	LPS directly activates the inflammatory enzyme caspase-11 and indirectly activates NF-κB	Causes pyroptosis, recruits macrophages, activates hepatic stellate cells, and causes liver fibrosis and even liver death
Steatosis	Accumulation of FFAs that binds to TLRs and activate the C-JNK and NF-κB pathways	Destroys the liver cell membrane, up-regulates MCP-1 expression, recruits macrophages and promotes lipid synthesis
Oxidative stress	Excessive ROS activates Kupffer cells	Cells secrete cytokines and participate in the inflammatory response; UCP-2 expression is upregulated
Fibrosis	Increased lipid peroxides and ApoB100 hydrolysis	Cells secrete TGF-β1 to activate hepatic stellate cells and aggravate fibrosis

**Table 2:** Therapeutic approaches targeting Kupffer cells in individuals with liver diseases

Categorization	Subsets	Main findings	Ref
Activation of Kupffer cells	Benzyl isothiocyanate	Inhibits cholesterol crystal-activated NLRP3 inflammasomes in Kupffer cells to improve high-fat/high-cholesterol induced changes in the liver	Chen et al., (2020)
	Alagebrium	Increases AGEs clearance and indirectly inhibits hepatic stellate cell activation	Fernando et al., (2019)
	Resveratrol	Improvs the inflammation and fibrosis by inhibiting LPS reactivity controlled by CD14 expression in Kupffer cells	Takaomi et al., (2016)
M1 and M2 Kupffer cells polarization	β-Cryptoxanthin	Prevents and reverses insulin resistance and steatohepatitis	Ni et al., (2015)
	Astaxanthin	Regulates macrophage/Kupffer cells polarization and hepatic homeostasis	Ni et al., (2016)
	RORα	Enhances M2 polarization in Kupffer cells	Han et al., (2017)
	Liraglutide	Regulates the M2 polarization of Kupffer cells to reduce liver steatosis	Li et al., (2019)
	Saxagliptin	Regulates the polarization of M1 / M2 macrophages / Kupffer cells	Yang et al., (2018)

## Abbreviations

NAFLD: non-alcoholic fatty liver disease
FDA: Food and Drug Administration
HCC: hepatocellular carcinoma
NAFL: non-alcoholic fatty liver
NASH: non-alcoholic steatohepatitis
T2DM: type 2 diabetes mellitus
IR: insulin resistance
CVD: cardiovascular disease
MetS: metabolic syndrome
SR: scavenger receptor
IBA-1: ionized calcium binding adapter molecule 1
CLEC‐4F: C‐type lectin domain family 4 member F
ATM: adipose tissue macrophages
NLRP3: Nod-like receptor protein 3
HFD: high fat diet
CXCL1: C‐X‐C motif chemokine ligand 1
MCD: methionine-choline deficient
TLR: Toll-like receptor
LPS: lipopolysaccharide
NF-κB: nuclear factor-kappa B
MAPK: mitogen-activated protein kinase
ERK1: extracellular signal-regulated kinase 1
JNK: Jun N-terminal kinase
IRF3: interferon regulatory factor 3
iNOS: inducible nitric oxide synthase
TNF: tumor necrosis factor
IL: interleukin
ROS: reactive oxygen species
ALD: alcoholic liver disease
IFN-γ: interferon-γ
MyD88: myeloid differentiation molecular 88
TRAFs: TNFR-associated factors
IRAKs: IL-1 receptor-associated kinases
TGF: transforming growth factor
TAK1: TGF-beta-activated kinase 1
TRAF3: TNF receptor associated factor 3
GSDMD: gasdermin D
CuMF: marginal-copper high-fructose diet
FFAs: free fatty acids
SPIO: superparamagnetic iron oxide
MCP-1: macrophage chemotactic protein 1
AP-1: activating protein 1
Fas: fatty acid synthase
oxLDL: oxidized low-density lipoprotein
HMGB-1: high mobility group box 1
NADPH: nicotinamide adenine dinucleotide phosphate
UCP2: uncoupling protein 2
NKT: natural killer T
TβR: TGF-β1 receptor
AST: alanine aminotransferase
ALT: aspartate aminotransferase
GGT: gamma-glutamyl transpeptidase
NMR: nuclear magnetic resonance
BITC: Benzyl isothiocyanate
AGEs: advanced glycation end products
RSV: resveratrol
RORα: retinoic acid-associated orphan receptor α
